# Effects of folic acid supplementation on chronic atrophic gastritis based on MTHFR C677T polymorphism

**DOI:** 10.1097/MD.0000000000033980

**Published:** 2023-06-16

**Authors:** Siya Kong, Guoxin Zhang, Zhen Yang, Zihao Kong, Feng Ye

**Affiliations:** a Department of Gastroenterology, The First Affiliated Hospital of Nanjing Medical University, Nanjing, China; b First Clinical Medical College of Nanjing Medical University, Nanjing, China.

**Keywords:** atrophic gastritis, folic acid, *Helicobacter pylori*-negative, incisura, MTHFR C677T

## Abstract

It has been shown the methylenetetrahydrofolate reductase (MTHFR) 677TT (rs 1801133) genotype predicts histopathological alterations in the incisura of patients with chronic atrophic gastritis (CAG). MTHFR is a crucial enzyme in fatty acid (FA) metabolism. This study aimed to evaluate the influence of FA supplementation in CAG patients without *Helicobacter pylori* infection and the MTHFR C677T (rs 1801133) genotype as a potential CAG predictor.

**Methods:**

A total of 96 CAG patients, aged 21 to 72 years old, were enrolled in this study. After 6 months of treatment, histopathological outcomes were compared among patients treated with weifuchun (WFC) (1.44 g 3 times per os per day), those treated with WFC and FA (5 mg once daily), and those treated with WFC, FA, and vitamin B12 (VB12) (0.5 mg 3 times per day) based on the Operative Link on Gastritis/Intestinal Metaplasia assessment staging systems.

**Results:**

Atrophic lesions in patients treated with WFC and FA improved more than in patients treated only with WFC therapy (78.1% vs 53.3%, *P* = .04). Atrophic or intestinal metaplasia (IM) lesions in the incisura of patients with the TT genotype were better than those in patients with the CC/CT genotype (*P* = .02).

**Conclusion:**

The treatment of CAG patients with 5 mg of FA supplements daily for 6 months improved their gastric atrophy status, especially for the Operative Link on Gastritis/Intestinal Metaplasia assessment stages I/II. Moreover, our study is the first to reveal that patients with the MTHFR 677TT genotype require more timely and effective FA treatment than those with the CC/CT genotype.

## 1. Introduction

Chronic atrophic gastritis (CAG), a common precancerous lesion of stomach cancer, is characterized by the loss of gastric glands and is associated with the development and progression of gastric cancer.^[1]^ The updated Sydney System is a guideline used for the classification and grading of CAG histopathology and for evaluating the treatment efficacy of CAG.^[2]^ The Operative Link on Gastritis/Intestinal Metaplasia Assessment staging system distinguishes low, medium, and high risk for gastric cancer and divides patients into 5 subgroups (0–IV).^[3]^ The higher the stage, the higher the possibility that patients will develop gastric cancer based on the precancerous lesion.

One-carbon metabolism donors, which are used in DNA synthesis, repair, methylation, and regulation of gene expression, play an important role in gastric tumorigenesis.^[4]^ In particular, previous studies have reported an association between 1-carbon metabolism donors such as folic acid (FA) or vitamin B12 (VB12) and gastrointestinal cancer.^[5,6]^ However, it is not clear whether there is a direct connection between FA and VB12 supplementation dose and CAG.

It has been shown that 5,10-methylenetetrahydrofolate reductase (MTHFR), which is a crucial enzyme, participates in the production of methyl donors during methionine synthesis.^[7]^ In addition, it has been reported that its reduced activity and consequently DNA hypomethylation may increase the risk of gastric cancer.^[8,9]^ However, this can potentially be overcome by supplementation with 1-carbon metabolism donors, such as FA and VB12, due to interactions between MTHFR C677T and the vitamin B family.^[10]^ In our previous study, we demonstrated that *Helicobacter pylori*-negative CAG patients with the MTHFR 677TT genotype had more significant histopathological alterations in the incisura than patients with the MTHFR 677CC and CT genotypes according to the OLGA and OLGIM stages.^[11]^ More interestingly, literature reports suggest a possible association between the MTHFR C677T polymorphism and folate deficiency.^[12,13]^ Therefore, FA supplementation may be useful for better CAG treatment.

Traditional Chinese medicines, such as Weifuchun (WFC), are recommended for the treatment of CAG and have been shown to be effective for improving atrophic gastritis and intestinal metaplasia (IM) by consensus reports.^[14,15]^ WFC, a well-known drug used in traditional Chinese medicine, is an effective intervention in patients with CAG.^[14]^ It is composed of 3 core ingredients: Panax ginseng C. A. Meyer, Citrus aurantium L., and Rabdosia amethystoides (Benth.)Hara. (Supplementary Table S1, http://links.lww.com/MD/J108), respectively.^[14]^ A previous study employed ultra-high-performance liquid chromatography coupled with electrospray ionization quadrupole time-of-flight mass spectrometry and network pharmacology to survey the major constituents of WFC.^[16]^ A total of 178 compounds were identified in WFC, including 93 compounds (70 terpenes) originally from Rabdosia amethystoides, 51 compounds from Citrus aurantium L., and 31 compounds from Panax ginseng C. A. Meyer, which demonstrated the material basis of WFC. Thirteen active components, 48 therapeutic targets, and 61 pathways were found to treat precancerous lesions of gastric cancer based on the main compounds in WFC, their metabolites in rat plasma, and existing databases.^[16]^ It had reported the molecular mechanisms of WFC to treat gastritis in vitro or in vivo, including regulation of NF-κB, MAPK, RUNX3/TGF-beta/Smad, Hedgehog and Wnt signaling pathways, modulation of the expression of oncogenes and tumor suppressor genes.^[14,17]^ In this study, we investigated the influence of FA supplementation on the treatment of CAG patients without *H pylori* infection, and whether the MTHFR C677T polymorphism status might be useful as a potential predictor for the clinical treatment of CAG.

## 2. Patients and methods

### 2.1. Patients and treatments

For the purpose of this study, subjects who underwent esophagogastroduodenoscopy to evaluate the cause of their abdominal symptoms or for gastric cancer screening at the First Affiliated Hospital of Nanjing Medical University from January 2019 to January 2020 were recruited. Based on the initial diagnosis, patients were recruited to receive (for the first time) treatment for CAG. The inclusion criteria were patients with CAG aged between 18 and 75 years. The exclusion criteria were as follows: *H pylori*-positive status (an independent contributing factor to the development of CAG); previous *H pylori* eradication; and intake of antibiotics, FA, VB12, proton pump inhibitors, or H_2_-receptor blockers in the previous month.

This study was a randomized, open-label, single-center trial. The participants were randomly distributed into 3 groups to receive 6 months of treatment:^[18]^ WFC group (conventional treatment), in which patients were treated orally with WFC therapy (1.44 g, 3 times per day)^[18]^; WFC + FA group, in which patients were treated orally with WFC (1.44 g, 3 times per day) and FA (5 mg, once per day)^[19–21]^; and WFC, FA, and VB12 groups, in which patients were treated orally with a combination of WFC (1.44 g, 3 times per day), FA (5 mg, once per day), and VB12 (0.5 mg) 3 times per day.^[22]^ All medications were administered after the meals. Prior to initiating treatment, baseline characteristics of the study participants were collected, including age, sex, family history, smoking habits, alcohol consumption, and body mass index. The patient characteristics are presented in Table 1. *H pylori* infection was assessed using the ^13^C-urea breath test. Before and after treatment, endoscopy and biopsy were performed separately for all patients in the cohort. The participants were interviewed about their abdominal symptoms during follow-up every month. The study protocol was reviewed and approved by the Ethics Committee of the First Affiliated Hospital of Nanjing Medical University. Written informed consent was obtained from all the patients in the cohort. This trial was completed and registered with ClinicalTrials.gov (ChiCTR1900020815, Chinese Clinical Trial Registry) on 20/01/2019. The study was conducted in accordance with the ethical standards of the institutional research committee and the 1964 Helsinki Declaration and its later amendments or comparable ethical standards.

**Table 1:** Baseline characteristics of WFC, WFC + FA and WFC + FA + VB12 groups.

Characteristic	WFC group (n = 30)	WFC + FA group (n = 32)	WFC + FA + VB12 group (n = 28)	*P* value
Age (yr)	53 ± 9	53 ± 11	54 ± 9	*P* = .54^a^
Male, n (%)	12 (40.0%)	18 (56.3%)	11 (39.3%)	*P* = .32^b^
Family history, n (%)	11 (36.7%)	20 (62.5%)	14 (50%)	*P* = .13^b^
Smoking status				*P* = .51^b^
Never	24 (80.0%)	22 (68.8%)	19 (67.9%)	
Current/Former	6 (20.0%)	10 (31.3%)	9 (32.1%)	
Alcohol status				*P* = .53^b^
Never	22 (73.3%)	27 (84.4%)	21 (75.0%)	
Current/Former	8 (26.7%)	5 (15.6%)	7 (25.0%)	
BMI (kg/m^2^)	22.8 ± 3.0	22.0 ± 2.8	22.5 ± 2.5	*P* = .55^a^
OLGA stage				*P* = .39^b^
I	15 (50.0%)	13 (40.6%)	14 (50%)	
II	12 (40.0%)	11 (34.4%)	5 (17.9%)	
III	3 (10.0%)	7 (21.9%)	9 (32.1%)	
IV	0 (0.0%)	1 (3.1%)	0 (0.0%)	
OLGIM stage				*P* = .82^b^
I	7 (23.3%)	8 (25.0%)	8 (28.6%)	
II	9 (30.0%)	9 (28.1%)	9 (32.1%)	
III	14 (46.7%)	13 (40.6%)	10 (35.7%)	
IV	0 (0.0%)	2 (6.3%)	1 (3.6%)	
MTHFR C677T genotype	.			*P* = .69^b^
CC + CT	24 (35.8%)	23 (34.3%)	20 (29.9%)	
TT	6 (26.1%)	9 (39.1%)	8 (34.8%)	

Data are presented as number (%) or mean ± SD (standard deviation).

BMI = body mass index, FA = folic acid, OLGA = The Operative Link on Gastritis assessment: a system that divides patients into 5 subgroups (0–IV), the higher the stage, the higher the likelihood that patients will develop gastric cancer based on the precancerous lesion, OLGIM = The Operative Link on Gastritis Intestinal Metaplasia assessment, VB12 = vitamin B12.

a Based on ANOVA test.

b Based on Pearson chi-squared test.

### 2.2. CAG assessment and grading

All 5 or more endoscopic biopsies were obtained from the greater and lesser curvatures of the corpus, greater and lesser curvatures of the antrum, and incisura before and after the treatment performed by the authors (F.Y. and G.Z.) via upper gastrointestinal endoscopy. Histopathological outcomes were reviewed by 2 independent pathologists in our hospital following the updated Sydney System.^[23]^ The histopathological data were obtained from patients’ medical records. Additionally, the authors evaluated the gastritis stage based on the OLGA and OLGIM staging systems.^[24]^

Good and poor responders were defined according to changes in OLGA and OLGIM stages before and after the corresponding treatment. For example, if the OLGA stage was IV before therapy and I after therapy, the change was recorded in 3 units. A positive sign indicated an improvement in the histopathological findings after therapy; no change was designated as 0, and a negative sign indicated worsening of the histopathological status after therapy. Hence, good responders were designated with positive numbers (1, 2, and 3), while poor responders were designated with 0 or negative numbers (−3, −2, −1, and 0).

### 2.3. Serological testing for plasma folate, pepsinogen, and homocysteine levels

Plasma levels of folate, pepsinogen, and homocysteine were determined according to the procedure described in our previous study^[11]^ before and after treatment completion. Venous blood samples (10 mL) were collected from each patient in EDTA-containing tubes. The absorbance of pepsinogen was measured at 450 nm using an ELISA kit. High-performance liquid chromatography was used to measure the homocysteine levels. A radioimmunoassay was used to measure plasma folate levels.

### 2.4. DNA extraction and MTHFR polymorphism genotyping

Genomic DNA was extracted from blood samples using a column extraction kit (QIAGEN Inc., Valencia, CA). Digital fluorescence molecular hybridization (DFMH) was performed using a commercial kit (Sino Era Genotech, Beijing, China) to detect MTHFR 677C > T rs 1801133 polymorphism. This procedure was performed as previously described.^[25]^

### 2.5. Statistical analysis

Analysis of variance or Pearson chi-squared test was used to compare baseline characteristics among the groups. The rank-sum test was applied to compare OLGA and OLGIM stages before and after treatment in all groups. A paired *t* test was used to compare the levels of plasma folate, pepsinogen, and homocysteine before and after treatment. Pearson chi-squared test was used to compare pathological changes in patients before and after therapy. The results were considered statistically significant if the *P* value was <.05. IBM SPSS version 25.0 (SPSS Inc., Chicago, IL) was used for statistical analysis.

## 3. Results

### 3.1. General clinical information

**Figure 1.**
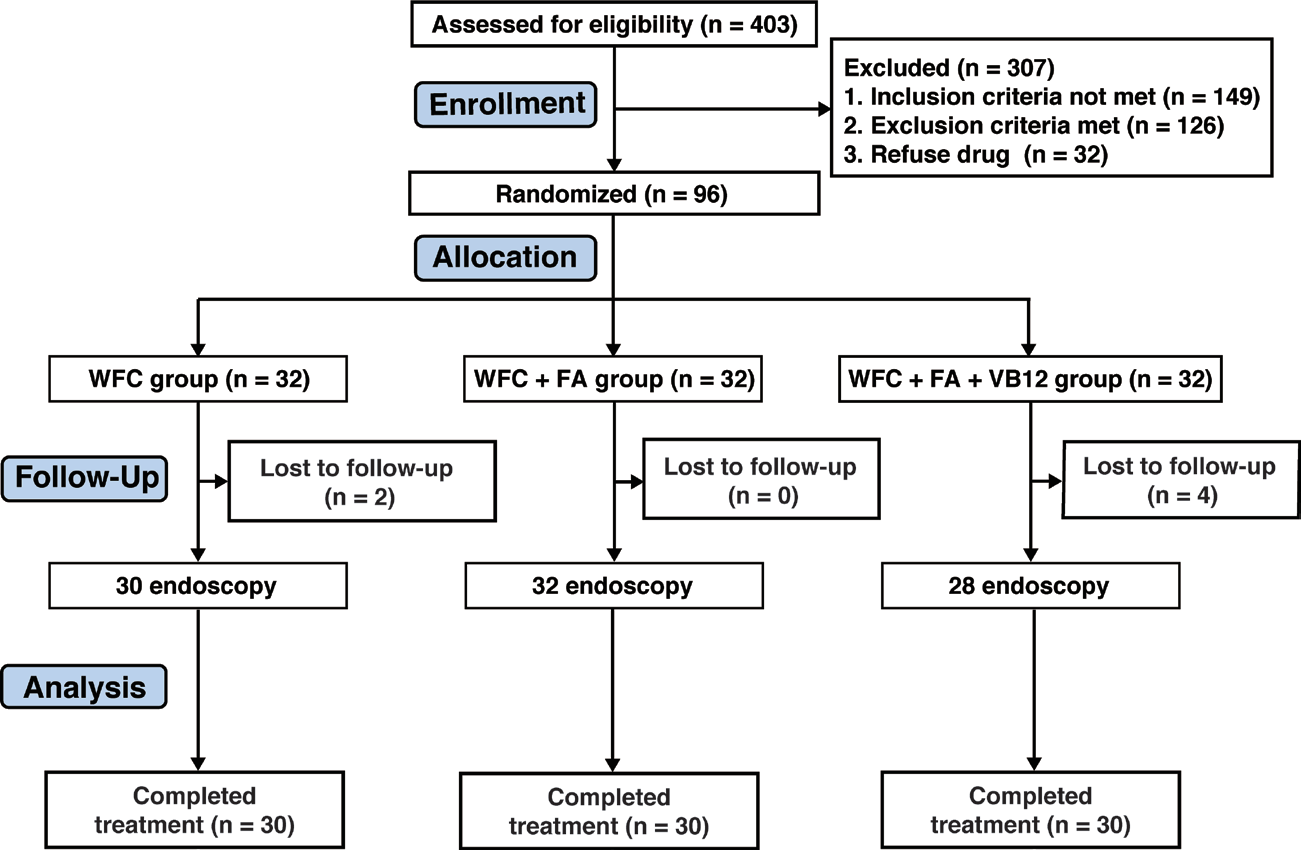
Flow chart of the study design. WFC = weifuchun (1.44 g 3 times per os per day), FA = folic acid (5 mg once daily), VB12 = vitamin B12 (0.5 mg 3 times per day). Treatment duration: 6 mo. Six cases were withdrawn for losing connection.

A total of 403 participants were screened for potential eligibility to participate in the study through an initial interview. Of these, 275 were excluded for the following reasons: *H pylori*-positivity (n = 58), previous *H pylori* eradication (n = 41), intake of additional drugs (n = 27), inadequate biopsy samples for evaluation (n = 63), diagnosis of gastric cancer (n = 5), or diagnosis of no atrophy (n = 81). Hence, 128 patients diagnosed with CAG for the first time were enrolled in this study. Thirty-two patients declined further follow-up visits, and the drugs were included in the trial. The remaining 96 patients, ranging in age from 21 to 72 years, were randomly allocated into 3 groups: the WFC group (n = 32), the WFC + FA group (n = 32), and the WFC + FA + VB12 group (n = 32). Six patients were lost to follow-up; therefore, the prospective trial consisted of 90 CAG patients (shown in Fig. 1) who underwent endoscopy after 6 months to evaluate the efficacy of treatment.

The baseline characteristics of the study participants (age,sex, family history, smoking and alcohol consumption, OLGA stage, OLGIM stage, MTHFR C677T genotype, body mass index) are presented in Table 1. In our study, 67 (74.4%) patients presented with the MTHFR 677 CC or CT genotype, whereas the TT genotype was detected in 23 patients (25.6%). The observed MTHFR C677T genotype distribution was consistent with previously reported data in the Chinese population.^[26]^ Ten percent of patients in our study had corpus atrophic gastritis, and 86.7% of patients had antrum atrophic gastritis. No differences in OLGA and OLGIM stages were observed among the 3 groups (OLGA, *P* = .39; OLGIM, *P* = .82, Kruskal–Wallis rank-sum test). A downgrade in OLGA and OLGIM stages was observed 6 months after treatment in all groups, that is, the histopathology results for all participants included in the study improved (*P* < .05, Wilcoxon rank-sum test).

**Figure 2.**
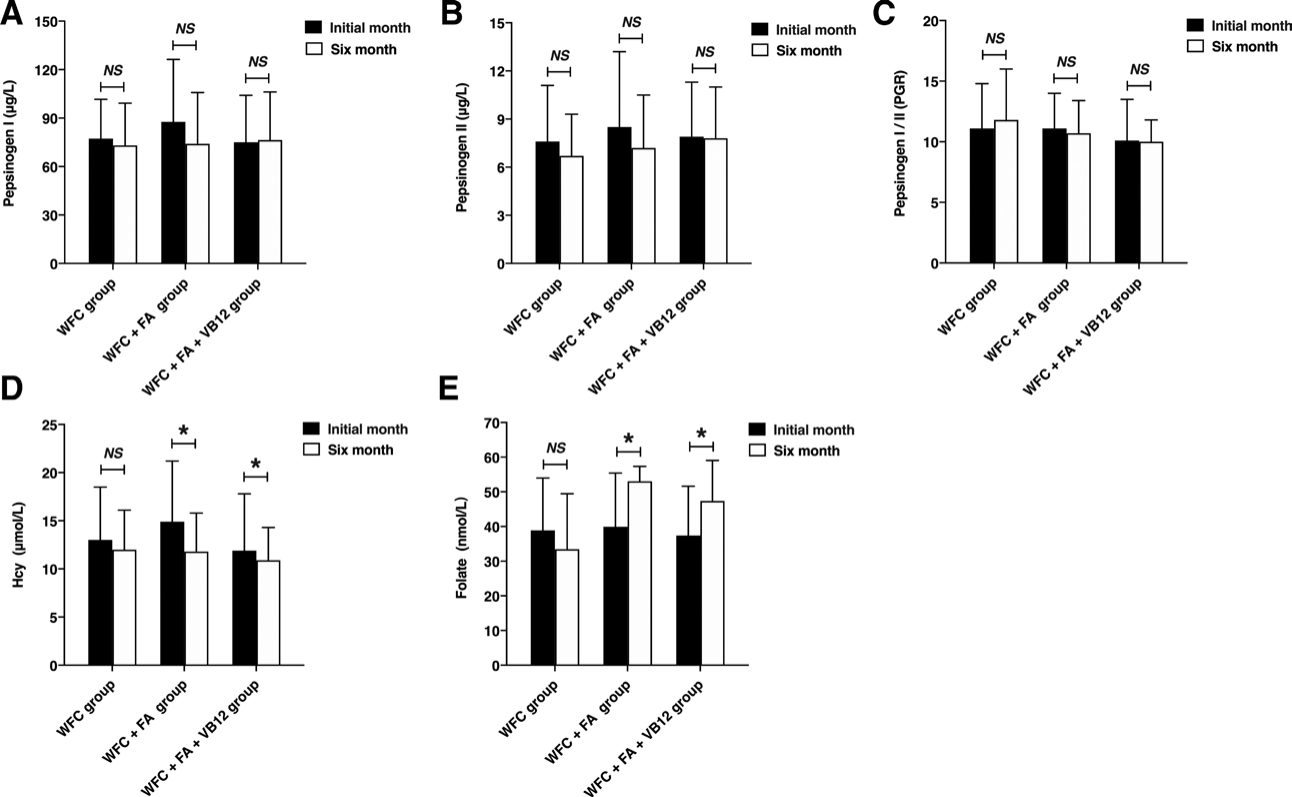
Serological testing for pepsinogen I, pepsinogen II, pepsinogen I/II (PGR), homocysteine levels, and folate. *P ≤ .05, NS = not significant. A paired *t* test was used for the statistical analysis. WFC = weifuchun.

As shown in Figure 2, no significant difference was observed in pepsinogen I, pepsinogen II, or pepsinogen I/II expression 6 months after the initial treatment (*P* > .05). Homocysteine levels 6 months after treatment with FA or FA + VB12 were lower than those before treatment (WFC + FA group: *P* = .05; WFC + FA + VB12 group: *P* = .04). This phenomenon may be due to an increased level of folate following treatment (WFC + FA group: *P* = .01; WFC + FA + VB12 group: *P* = .03).

### 3.2. FA supplementation improved the histopathological status of CAG patients

A comparison of OLGA and OLGIM stages before and after therapy is presented in Table 2. Patients in the WFC + FA group showed improved histopathological status compared to those in the WFC group (78.1% vs 53.3%, *P* = .04) according to the OLGA staging system. However, no difference in the OLGA stage was observed in the WFC + FA + VB12 group compared to the WFC group, as well as between the 2 types of treatment (OLGA: WFC + FA + VB12 group vs WFC group, *P* = .26; WFC** + **FA group vs WFC** + **FA + VB12 group, *P* = .37). When the patients were stratified according to the OLGIM stage, no difference between either treatment and control was observed, nor between the 2 types of treatment (WFC + FA + VB12 group vs WFC group *P* = .12; WFC** + **FA group vs WFC group *P* = .25; WFC** + **FA group vs WFC** + **FA + VB12 group, *P* = .32).

**Table 2:** Comparison of the patients’ OLGA and OLGIM stages before and after therapy.

	OLGA		OLGIM	
	Poor responder	Good responder	Poor responder	Good responder
WFC group	14 (46.7%)	16 (53.3%)	8 (26.7%)	22 (73.3%)
WFC + FA group	7 (21.9%)	25 (78.1%)	13 (40.6%)	19 (59.4%)
WFC + FA + VB12 group	9 (32.1%)	19 (67.9%)	13 (46.4%)	15 (53.6%)

Using Pearson chi-squared test, the comparison of patients’ OLGA stages before and after therapy is as follows: WFC + FA group vs WFC group, 78.1% vs 53.3%, *P* = .04; WFC + FA + VB12 group vs WFC group, 67.9% vs 53.3%, *P* = .26; WFC + FA group vs WFC + FA + VB12 group, 78.1% vs 67.9%, *P* = .37. The comparison of patients’ OLGIM stages before and after therapy is as follows: WFC + FA + VB12 group vs WFC group, *P* = .12; WFC + FA group vs WFC group, *P* = .25; WFC + FA group vs WFC + FA + VB12 group, *P* = .32.

FA = folic acid, OLGA = The Operative Link on Gastritis assessment, OLGIM = The Operative Link on Gastritis Intestinal Metaplasia assessment, VB12 = vitamin B12, WFC = weifuchun; Good responder indicates an improvement in the histopathology findings after therapy. Poor responder indicates no change or a worsening (negative sign) of the histopathological status after therapy.

### 3.3. Patients with the MTHFR C677T TT genotype showed better improvements in incisura histopathological alterations after treatment compared to patients with the CC or CT genotype

**Figure 3.**
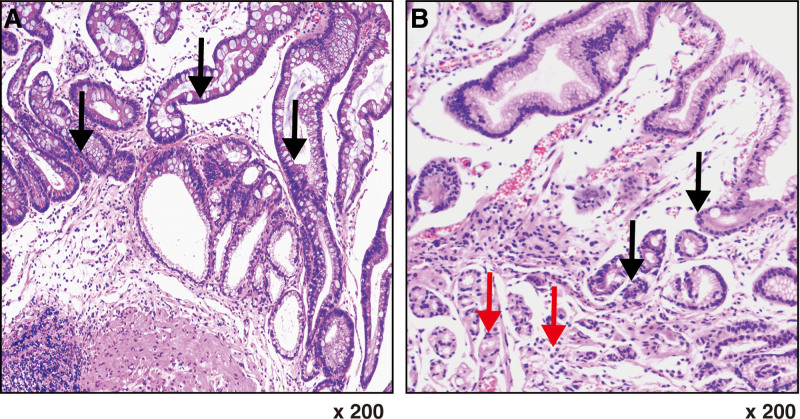
The improvement of atrophic and IM incisura histopathological alterations after therapy in patient No.63 with MTHFR 677TT. (A) Histopathology before treatment (200× magnification). It shows severe intestinal metaplasia of the gastric mucosal glandular epithelium, reduced inherent glandular structures, and atrophy. There was notable inflammatory cell infiltration in the background. Black arrows indicate intestinal metaplasia. (B) Histopathology after treatment (200× magnification). It revealed focal intestinal metaplasia of the gastric mucosal glandular epithelium, regeneration of the inherent glandular structures, and no significant atrophic changes. Scattered inflammatory cell infiltration was observed in the background. Black arrows indicate focal intestinal metaplasia, while red arrows indicate the regeneration of inherent glandular structures without significant atrophic changes. IM = intestinal metaplasia, MTHFR = methylenetetrahydrofolate reductase.

There was no significant difference in treatment regimens between patients with the CC or CT genotype and those with the TT genotype. Greater improvements in the histopathology results for both atrophy and IM lesions on the incisura (represented by a change in histopathological alterations in the same patients according to the updated Sydney system) were also observed in patients with the TT genotype than in those with the CC or CT genotypes after treatment in the WFC, WFC + FA, and WFC + FA + VB12 groups (*P* = .02) (Table 3 and Fig. 3).

**Table 3:** The status of atrophic and IM incisura histopathological alterations in patients after treatment in the 3 groups stratified according to the MTHFR C677T genotype and updated Sydney system.

MTHFR C677T genotype	Atrophy			IM		
	Poor responder	Good responder	*P* a	Poor responder	Good responder	*P* a
CC + CT	54 (80.6%)	13 (19.4%)	.02	48 (71.6%)	19 (28.4%)	.02
TT	13 (56.5%)	10 (43.5%)		10 (43.5%)	13 (56.5%)	

IM = intestinal metaplasia.

a Based on chi-square test. Good responder indicates an improvement in the histopathology findings after therapy. Poor responder indicates no change or a worsening (negative sign) of the histopathological status after therapy.

## 4. Discussion

With the improvement in the cure rate for *H pylori*, scientific attention has turned to the treatment of *H pylori*-negative CAG and gastric cancer. In China, FA and VB12 have long been a part of the clinical treatment regimen for gastric atrophy.^[20,27]^ However, the beneficial effects of this therapy were not observed in all the patients.^[20,28]^ Additionally, among premenopausal women, a higher FA intake was associated with a 2.62-fold risk of stomach cancer.^[29]^ In this prospective study, we confirmed that treatment with 5 mg of FA daily for 6 months is effective in improving gastric atrophy. However, the same effect was not observed in patients with IM when FA or a combination of FA and VB12 was used. It seems that in our study, FA lost its efficacy against atrophy when taken together with VB12. These findings are inconsistent with previous findings reported in the literature^[19,20]^ and this discrepancy may be explained by several factors. First, the overuse of vitamin B as a methyl donor may have no benefits, since a high level of serum folate might be associated with low global methylation in some situations.^[30]^ It could be that VB12 promotes FA metabolism, which in turn leads to better use of FA with the same dose. As shown in Figure 2, the level of folate in the WFC + FA + VB12 group was lower, and the level of homocysteine was higher than that in the WFC + FA group with the same dosage of 5 mg of FA, which might indicate that more folate

was used for methylation metabolism. However, specific methylation metabolism may be associated with low global methylation, which might indicate the overuse of FA.^[30]^ Second, well-nourished patients are not recommended FA supplements, as previously reported.^[21]^ Third, it is difficult to determine a suitable dose and treatment duration based on the patient histopathology results. Thus, the contradictory findings in our study might be the result of an inadequate treatment duration. Indeed, it has been shown that histopathological status can improve after 1 year of FA and VB12 intake.^[20]^ Fourth, the appropriate timing for FA treatment according to the patient histological results remains unclear. Overall, our results indicate that the combination of FA (5 mg daily) and VB12 (0.5 mg 3 times per day) for 6 months may not be beneficial for patients with CAG.

WFC is a traditional Chinese medicine composed of Radix Ginseng Rubra (red ginseng), Rabdosia amethystoides H. Hara, and fried Fructus Aurantii,^[14]^ which dramatically improves clinical outcomes in CAG patients via antioxidant effects, improving gastric mucosal blood flow,^[31]^ inducing apoptosis, inhibiting angiogenesis,^[32]^ blocking NF-κB pathways,^[17]^ and modulating the expression of oncogenes and tumor suppressor genes.^[14]^ However, to date, there has been no research on the effects of WFC on FA metabolism. As shown in Figure 2, there was no difference between FA concentrations before and after treatment in the WFC group. It appears that WFC might have had no effect on FA metabolism in our study.

In China, the MTHFR 677CC or CT genotype is found in 75% of the population.^[26]^ In our previous study, we showed that the TT genotype may be an appropriate predictor of the development of histopathological alterations in the incisura and thus may have clinical relevance.^[11]^ The histopathology of patients with the TT genotype was higher than that of patients with the CC and CT genotypes. Therefore, prompt treatment is necessary for patients with the TT genotype to achieve good CAG control.

This study had some limitations. First, it was difficult to clearly determine the efficacy of FA and VB12 treatment in the relatively small number of patients included in the study (n = 90). Only 10% of patients in our study had corpus atrophic gastritis. These results may be applicable to the early stages of CAG. Thus, more participants with more severe lesions are needed to reach evidence-based conclusions. Second, the efficacy of FA and VB12 treatment according to the MTHFR C677T genotype could not be examined because of the small number of patients with the TT genotype. Third, the patients were aware of what they were taking as treatment; therefore, the study was not completely double-blinded. Fourth, to verify the efficacy of FA and VB12 treatment, the best control group would be the one without any treatment. However, it would be immoral to leave patients with CAG without any therapeutic intervention, and conventional therapy was administered to the control group. Finally, we did not use a food survey questionnaire to assess the nutritional status of patients before the study. Consequently, the potential influence of nutritional status on observed outcomes cannot be excluded. Future research should consider incorporating food survey questionnaires to more effectively control nutritional status.

In summary, the combination of 5 mg FA with WFC daily for 6 months can be used to improve the histopathological status of *H pylori*-negative CAG compared to WFC alone according to the OLGA staging system. Such vitamin supplementation seemed harmless and did not require MTHFR genotyping to justify treatment within the scope of this test dose. Additionally, patients with the TT genotype and CAG receive greater benefits from timely and effective treatment than those with the CC or CT genotype. However, more participants in future studies are needed to obtain a more reliable conclusion.

## Author contributions

**Conceptualization:** Siya Kong, Guoxin Zhang, Feng Ye.

**Data curation:** Siya Kong, Guoxin Zhang, Feng Ye.

**Formal analysis:** Siya Kong, Zhen Yang, Feng Ye.

**Funding acquisition:** Guoxin Zhang, Feng Ye.

**Investigation:** Siya Kong, Guoxin Zhang, Feng Ye.

**Methodology:** Siya Kong.

**Project administration:** Guoxin Zhang.

**Software:** Zhen Yang.

**Supervision:** Guoxin Zhang, Feng Ye.

**Validation:** Siya Kong.

**Visualization:** Zhen Yang, Zihao Kong.

**Writing – original draft:** Siya Kong.

**Writing – review & editing:** Siya Kong, Feng Ye.

## Supplementary Material
