# Supplementary material for: Effects of folic acid supplementation on chronic atrophic gastritis based on MTHFR C677T polymorphism
Source: Medicine (Baltimore). 2023 Jun 16;102(24):e33980. doi: 10.1097/MD.0000000000033980 (PMC10270466; doi:10.1097/MD.0000000000033980)
Supplement: Supplementary file 1 [file medi-102-e33980-s001.pdf]

**Table S1. Ingredients of Weifuchun**

| | |
|---|---|
| Panax ginseng C.A.Meyer | Ginsenoside Rd $C_{48}H_{82}O_{18}$<br>Ginsenoside Rb1 $C_{54}H_{92}O_{23}$<br>Ginsenoside Rb2 $C_{53}H_{90}O_{22}$<br>Ginsenoside Rg1 $C_{42}H_{72}O_{14}$<br>Ginsenoside Rh8 $C_{36}H_{60}O_{9}$<br>Ginsenoside Rc $C_{53}H_{90}O_{22}$<br>Ginsenoside Rf $C_{42}H_{72}O_{14}$<br>Stigmasterol $C_{29}H_{48}O$<br>Ginsenoside Re $C_{48}H_{82}O_{18}$<br>Dnop $C_{24}H_{38}O_{4}$<br>Beta-Elemene $C_{15}H_{24}$ |
| Rabdosia amethystoides (Benth.) Hara | Amethystonal $C_{20}H_{28}O_{5}$<br>14-acetylumbrosin $C_{22}H_{30}O_{5}$<br>ursolic acid $C_{30}H_{48}O_{3}$ |
| Citrus aurantium L. | N-Methyltyramine-O-alpha-L-rhamnopyranoside $C_{15}H_{23}NO_{5}$<br>Narirutin $C_{27}H_{32}O_{14}$<br>Nobilin $C_{20}H_{26}O_{5}$<br>Obacunoic acid $C_{26}H_{32}O_{8}$<br>Sesguoiaflavone $C_{31}H_{20}O_{9}$<br>Sinigrin $C_{10}H_{17}NO_{9}S_{2}$<br>Suberenon $C_{14}H_{12}O_{4}$<br>Zederone $C_{15}H_{18}O_{3}$ |
